# Supplementary material for: Quantitative Analysis of Factors Regulating Angiogenesis for Stem Cell Therapy
Source: Biology (Basel). 2021 Nov 20;10(11):1212. doi: 10.3390/biology10111212 (PMC8614798; doi:10.3390/biology10111212)
Supplement: Supplementary file 1 [file biology-10-01212-s001.zip › original+supplementary/biology-1387408-supplementary - Copy.pdf]

Flavonoids

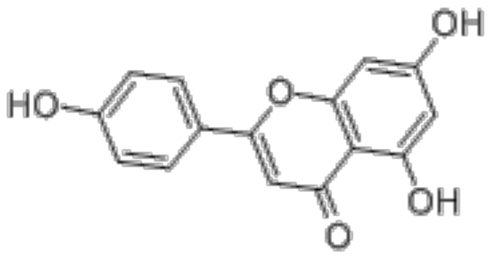

Apigenin

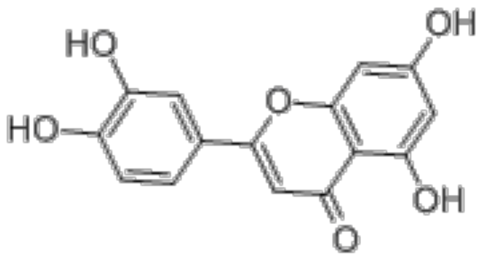

Luteolin

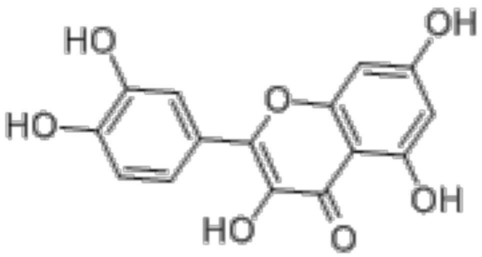

Quercetin

Polyphenol

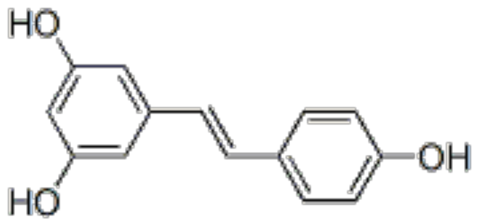

Resveratorol

Figure S1. Strucure of Flabonoids and Polyphenol used in this paper.

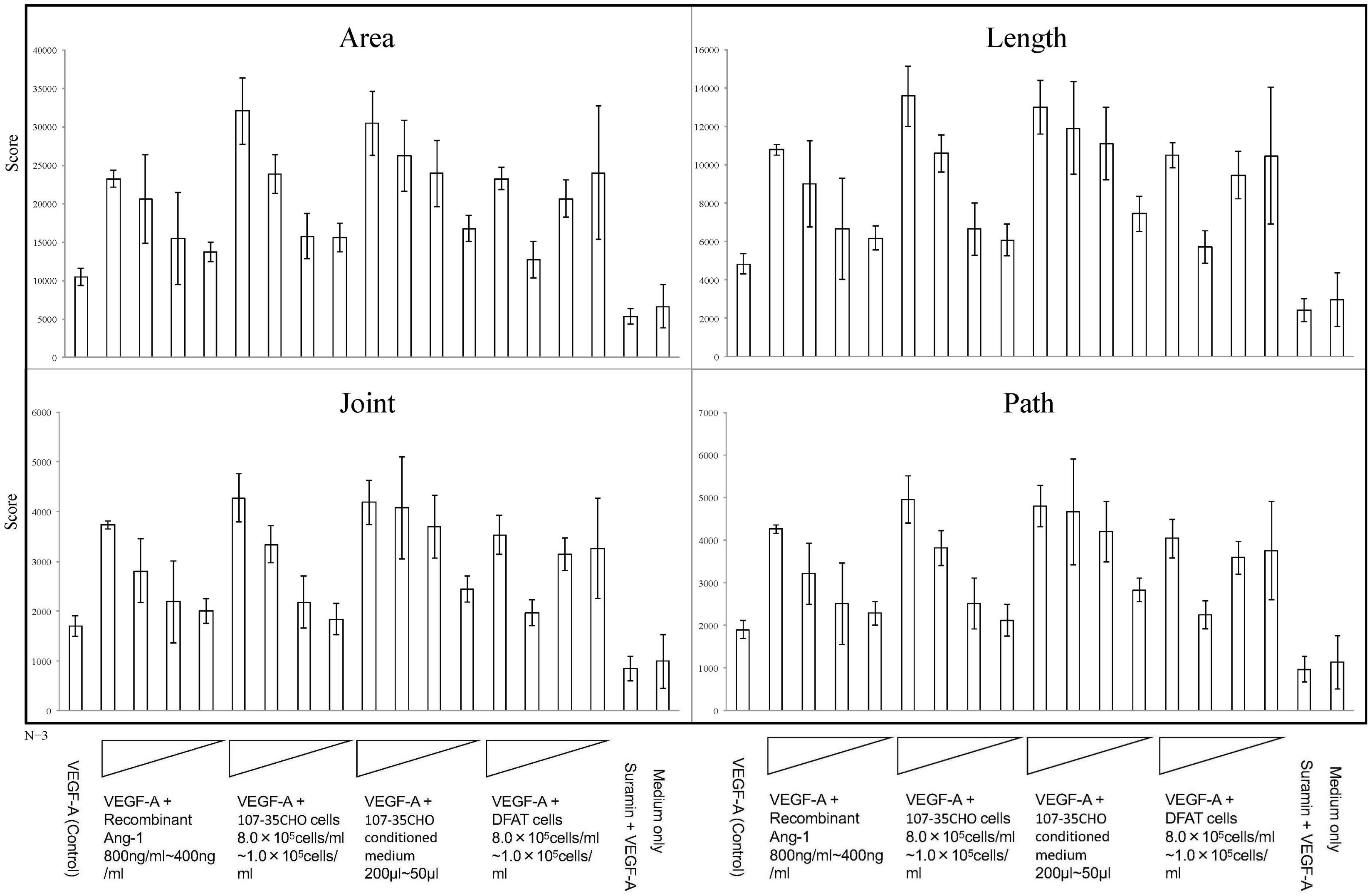

Figure S2. Results of quantitative analysis by using *KURABO Angiogenesis Image Analyzer*（Promotion） The promoting factor added to the cultures were Ang-1 that is known to stabilize the blood vessel. The results of quantitative analysis suggested that Ang-1 has a promoting effect on angiogenesis. Result of triplicated experiments for each sample were shown with standard deviations.

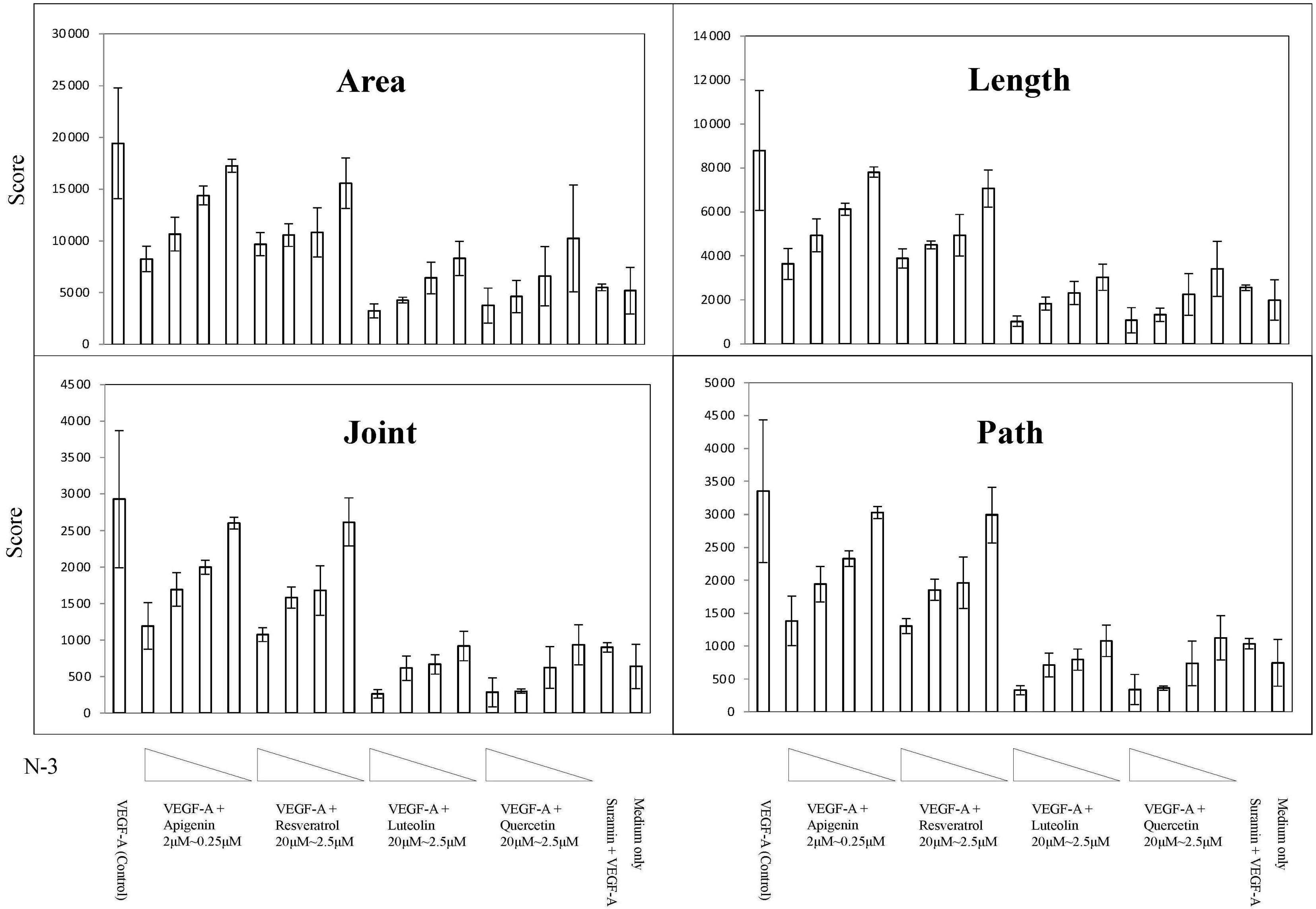

Figure S3. Results of quantitative analysis by using KURABO Angiogenesis Image Analyzer（Polyphenols）
The stained images were subjected to the quantitative microscope analysis by using the software attached to the kit. Flavonoids or polyphenol showed inhibitory effects on angiogenesis in a dose dependent manner. Particularly, luteolin and the quercetin showed remarkable effects. Result of triplicated experiments for each sample were shown with standard deviations.

Table S1. PCR primers for analyzed genes analyzed

Primer		Tm1	Tm2	
vWF	F	54	54	5'- AGC CTT GTG AAA CTG AAG CAT -3'
237 bp	R	58	54	5'- GCC CTG GTT GCC ATT GTA ATT C -3'
PECAM	F	58	55	5'- ACC GTG ACG GAA TCC TTC TCT -3'
246 bp	R	57	53	5'- GCT GGA CTC CAC TTT GCA C -3'
αSMA	F	57	55	5'- CTG AGC GTG GCT ATT CCT TC -3'
480 bp	R	60	55	5'- GCT GGA AGG TGG ACA GAG AG -3'
Flk1	F	57	55	5'- CAG CTT CCA AGT GGC TAA GG -3'
463 bp	R	51	48	5'- ATT TCC CAA ATG TTC CAC CA -3'
GAPDH	F	57	53	5'- ACC CAG AAG ACT GTG GAT GG -3'
421 bp	R	55	53	5'- CCC TGT TGC TGT AGC CAA AT -3'

Primer		Tm1	Tm2	
COX-1	F	55	51	5'- TCA TCG AGG AGT ACG TGC -3'
661 bp	R	57	53	5'- AGG GAC AGG TCT TGG TGT TG -3'
COX-2	F	53	53	5'- TAA ACT GCG CCT TTT CAA GG -3'
620 bp	R	51	48	5'- GTG ATA CTT TCT GTA CTG CT -3'
PPARγ	F	60	58	5'- CAG GAG CAG AGC AAA GAG GTG -3'
250 bp	R	56	53	5'- CAA ACT CAA ACT TGG GCT CCA -3'
GAPDH	F	57	53	5'- ACC CAG AAG ACT GTG GAT GG -3'
421 bp	R	55	53	5'- CCC TGT TGC TGT AGC CAA AT -3'

Primer		Tm1	Tm2	
eNOS	F	65	63	5'- GCT GCG CCA GGC TCT CAC CTT C -3'
105 bp	R	61	61	5'- GGC TGC AGC CCT TTG CTC TCA A -3'
GAPDH	F	57	53	5'- ACC CAG AAG ACT GTG GAT GG -3'
421 bp	R	55	53	5'- CCC TGT TGC TGT AGC CAA AT -3'
