# Supplementary figures and images for: Quantitative Analysis of Factors Regulating Angiogenesis for Stem Cell Therapy
Source: Biology (Basel). 2021 Nov 20;10(11):1212. doi: 10.3390/biology10111212 (PMC8614798; doi:10.3390/biology10111212)

RT-PCR original No.1

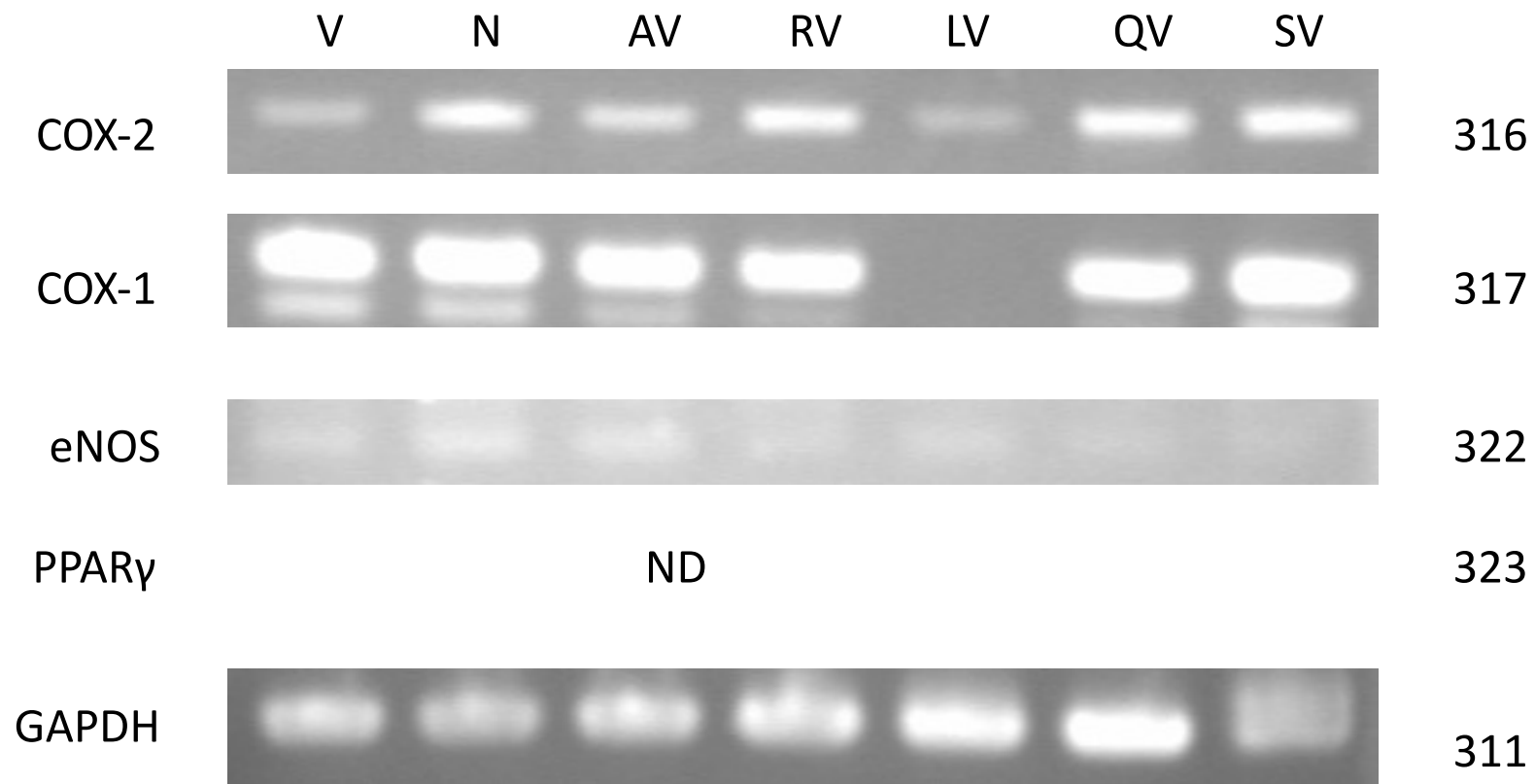

RT-PCR original No.2

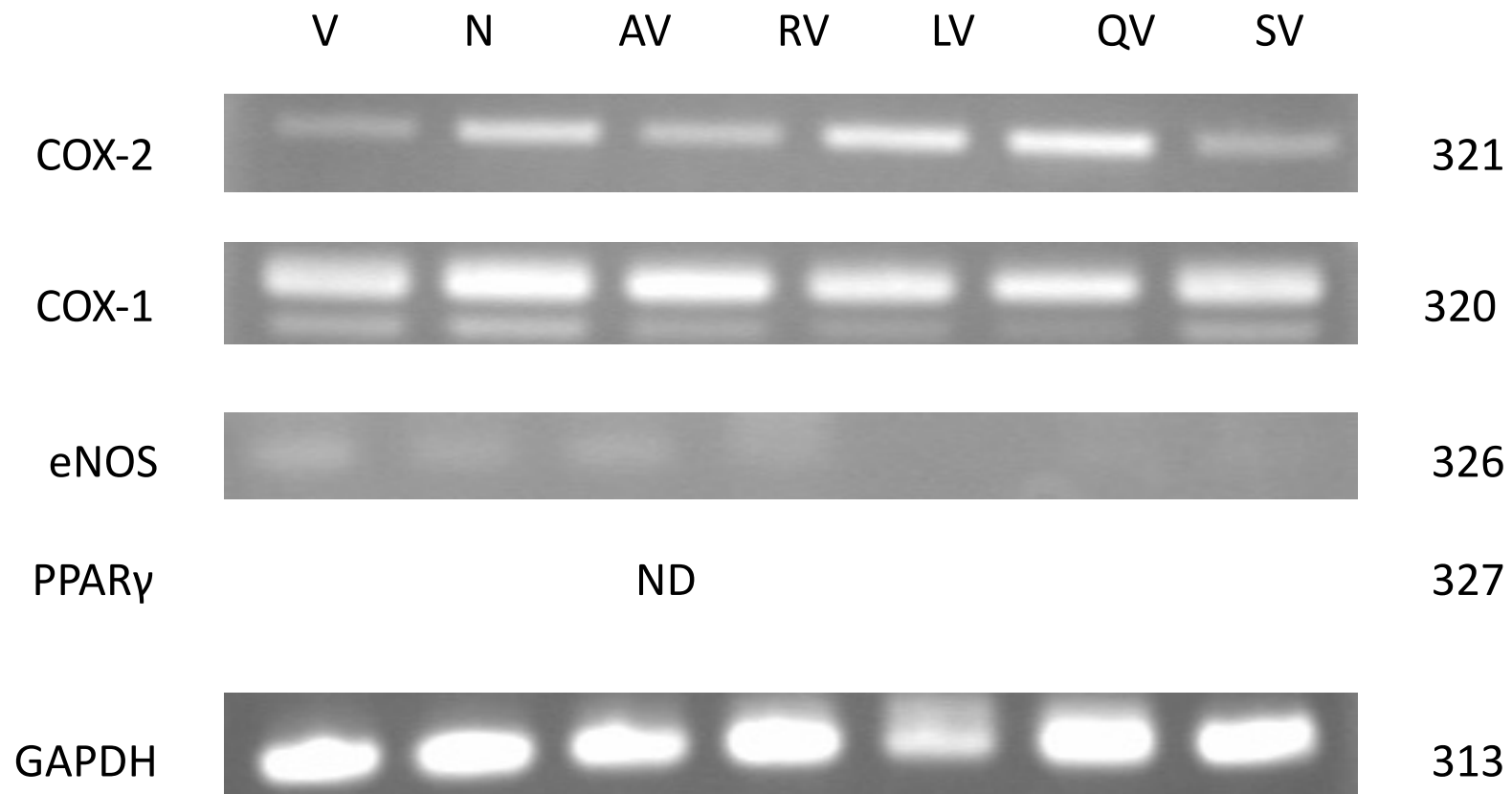

RT-PCR original No.3

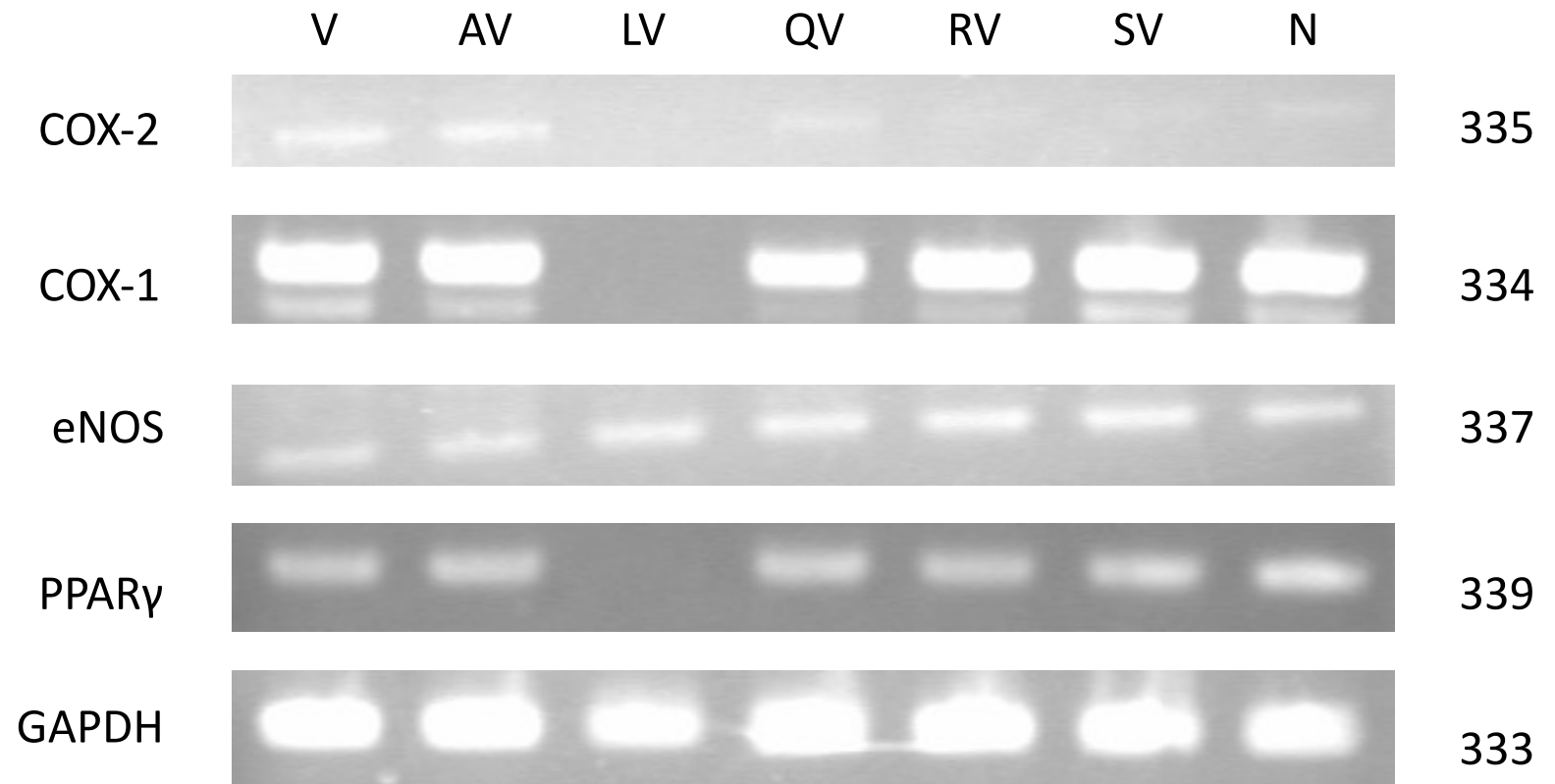

Supplement: Supplementary file 1 [file biology-10-01212-s001.zip › original+supplementary/biology-1387408-original images - Copy.pdf]
